# 14-3-3σ attenuates RhoGDI2-induced cisplatin resistance through activation of Erk and p38 in gastric cancer cells

**DOI:** 10.18632/oncotarget.1334

**Published:** 2013-10-19

**Authors:** In-Kyu Kim, Sun-Mi Park, Hee Jun Cho, Kyoung Eun Baek, In-Koo Nam, Seung-Ho Park, Ki-Jun Ryu, Jinhyun Ryu, Jungil Choi, Soon-Chan Hong, Jae Won Kim, Chang Won Lee, Sang Soo Kang, Jiyun Yoo

**Affiliations:** ^1^ Division of Applied Life Science/Research Institute of Life Science, Graduate School of Gyeongsang National University, Jinju, Korea; ^2^ Department of Anatomy and Neurobiology, Institute of Health Science, and Medical Research Center for Neural Dysfunction, School of Medicine, Gyeongsang National University, Jinju, Korea; ^3^ Department of Surgery, School of Medicine, Gyeongsang National University, Jinju, Korea

**Keywords:** RhoGDI2, 14-3-3σ, gastric cancer, chemoresistance, metastasis

## Abstract

Rho GDP dissociation inhibitor 2 (RhoGDI2) promotes tumor growth and malignant progression and enhances chemoresistance of gastric cancer. Recently, we noted an inverse correlation between RhoGDI2 and 14-3-3σ expression, which suggests that 14-3-3σ is a target of gastric cancer metastasis and the chemoresistance-promoting effect of RhoGDI2. Herein, we evaluated whether 14-3-3σ is regulated by RhoGDI2 and is functionally important for the RhoGDI2-induced cisplatin resistance of gastric cancer cells. We used highly metastatic and cisplatin-resistant RhoGDI2-overexpressing SNU-484 cells and observed decreased 14-3-3σ mRNA and protein expression. Depletion of 14-3-3σ in SNU-484 control cells enhanced cisplatin resistance, whereas restoration of 14-3-3σ in RhoGDI2-overexpressing SNU-484 cells impaired cisplatin resistance *in vitro* and *in vivo*. We also found that the phosphorylation levels of Erk and p38 kinases significantly decreased in RhoGDI2-overexpressing SNU-484 cells and recovered after 14-3-3σ expression, and that decreased activities of these kinases were critical for RhoGDI2-induced cisplatin resistance. In conclusion, 14-3-3σ is a RhoGDI2-regulated gene that appears to be important for suppressing the chemoresistance of gastric cancer cells.

## INTRODUCTION

Although the incidence and mortality of gastric cancer have steadily declined in recent decades, it remains the fourth most common type of cancer and the second leading cause of cancer mortality worldwide [1]. Surgery is an effective treatment for gastric cancer. Recent research has also shown that chemotherapy following radical surgery is an effective adjuvant therapy for East Asian patients [2]. Cisplatin is one of the most widely used drugs for chemotherapy and improves the overall survival for cancer patients [3-7]. However, cancer treatment is limited by the different efficacies of chemotherapeutic regimens, diverse disease states of patients and response rate to drugs, drug-related side-effects, acquisition of drug resistance, and cancer recurrence with metastasis [8-11]. To this end, the targeted approaches that focus on drug resistance-associated molecules are required to improve the efficacy of chemotherapy against advanced gastric cancers.

RhoGDI2 belongs to a family of Rho GTPase dissociate inhibitors (RhoGDIs). They are pivotal regulators of the function of Rho GTPase and typically exert their effects by forming a complex with Rho GTPase, and thereby modulating their nucleotide exchange and membrane association. Therefore, RhoGDIs play significant roles in regulating the actin cytoskeleton, cell polarity, microtubule dynamics, membrane transport pathways, and transcription factor activities [12, 13]. Unlike other members of the family (such as RhoGDI1 and RhoGDI3), RhoGDI2 is preferentially expressed in hematopoietic cells, and appears to have a narrow selectivity and lower binding affinity for Rho GTPases [14]. RhoGDI2 associates with and negatively regulates Rac1 and Rac3 in breast cancer cells, but not RhoA, Cdc42, and RhoC [15]. In contrast, it positively regulates Rac1 in human bladder cancer cells [16]. The significant role of RhoGDI2 in cancer has been previously noted in several lines of study. RhoGDI2 expression is inversely correlated with invasive capacity in bladder cancer cell lines [17]. Furthermore, reduced RhoGDI2 protein expression has been associated with poor prognosis for patients with advanced bladder cancer [18]. In contrast, RhoGDI2 mRNA expression is significantly higher in ovarian adenocarcinomas than in benign adenomas [19]. Consistent with this finding, RhoGDI2 is overexpressed in human breast cancer cell lines, and it increases cancer cell invasiveness and motility *in vitro* [20]. We also demonstrated that RhoGDI2 expression is positively correlated with tumor progression and metastatic potential in gastric cancer [21]. In addition, our recent work demonstrated that RhoGDI2 is associated with the acquisition of resistance to chemotherapeutic agents (such as cisplatin), which is a hallmark of aggressive cancers, in gastric cancer cells [22].

To delineate the mechanism by which RhoGDI2 promotes gastric cancer cell invasion and chemoresistance, we performed two-dimensional gel electrophoresis (2-DE) on proteins that were derived from a RhoGDI2-overexpressing SNU-484 human gastric cancer cell line and control cells, and noted that levels of 14-3-3σ, which is a member of the multifunctional 14-3-3 protein family, were significantly reduced [23]. In this study, we demonstrated that the downregulation of 14-3-3σ is largely correlated with the cisplatin-resistant phenotype of RhoGDI2-overexpessing gastric cancer cells. Of note, the restoration of 14-3-3σ is associated with impaired RhoGDI2-induced chemoresistance of gastric cancer cells through the activation of p38 and Erk.

## RESULTS

### RhoGDI2 downregulates 14-3-3σ expression

**Figure 1:**
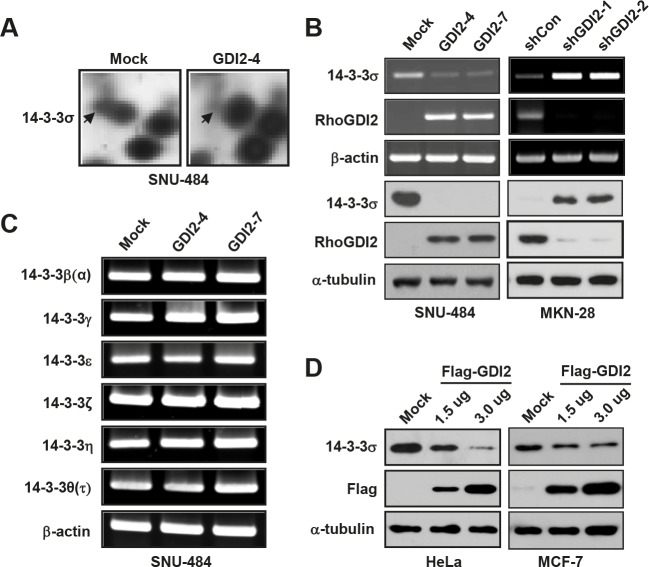
RhoGDI2 downregulates 14-3-3σ expression (A) Detailed 2-DE images of 14-3-3σ protein spots in RhoGDI2-overexpressing SNU-484(GDI2-4) cells compared with SNU-484(Mock) cells. (B) mRNA (upper panel) and protein (lower panel) expression of 14-3-3σ in RhoGDI2-overexpressing SNU-484(GDI2-4 and GDI2-7) cells. (C) mRNA expression of 14-3-3 isoforms in RhoGDI2-overexpressing SNU-484(GDI2-4 and GDI2-7) cells. (D) HeLa and MCF-7 cells were transiently transfected with RhoGDI2-expressing plasmid (Flag-RhoGDI2) and immunoblotted with indicated antibodies.

Previously, we identified 14 downregulated proteins in RhoGDI2-overexpressing SNU-484(GDI2-4) gastric cancer cells compared with control SNU-484(Mock) cells by using comparative 2-DE [23]. For further analysis, we selected 14-3-3σ that was previously implicated in cancer cell proliferation, metastasis, and apoptosis. To validate our mass spectrometry results, we performed reverse transcription-polymerase chain reaction and western blot analyses to determine the mRNA and protein expression levels of 14-3-3σ in RhoGDI2-overexpressing SNU-484 cells and RhoGDI2-depleted MKN-28 cells. Consistent with the results of 2-DE and imaging analysis (Fig. 1A), the mRNA and protein expression of 14-3-3σ were significantly downregulated in RhoGDI2-overexpressing SNU-484 cells and upregulated in RhoGDI2-depleted MKN-28 cells, compared to its expression in control cells (Fig. 1B). We also examined the mRNA expression levels of the other 14-3-3 isoforms (β, γ, ε, ζ, η, and τ) in RhoGDI2-overexpressing SNU-484 cells, but could not find any significant differences between these cells and the control cells (Fig. 1C). To further elucidate whether the decreased expression of 14-3-3σ is associated with RhoGDI2 expression, we observed 14-3-3σ expression levels in HeLa cells and MCF-7 cells after transient transfection with a Flag-tagged RhoGDI2 expression vector. As shown in Fig. 1D, transient expression of RhoGDI2 caused the markedly reduced expression of 14-3-3σ compared with the expression level in the vector-transfected control cells, which suggests that 14-3-3σ is a direct target of RhoGDI2.

### Depletion of 14-3-3σ expression enhances cisplatin resistance of gastric cancer cells

**Figure 2:**
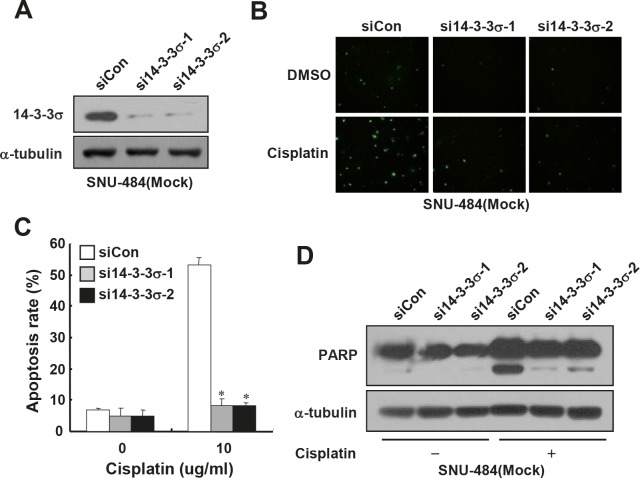
Depletion of 14-3-3σ expression enhances cisplatin resistance in gastric cancer cells (A) Representative immunoblot for 14-3-3σ in 14-3-3σ-depleted SNU-484(Mock) cells. (B) Representative images for TUNEL staining of 14-3-3σ-depleted SNU-484(Mock) cells after cisplatin treatment (10 μg/ml) for 24 h. (C) Histogram shows the ratio of TUNEL-positive 14-3-3σ-depleted SNU-484(Mock) cells after cisplatin treatment (10 μg/ml) for 24 h. Data are mean ± SD of three individual experiments, each in triplicate. *, *P* 0.01 as determined by paired Student *t* test. (D) Representative immunoblot for PARP cleavage after cisplatin treatment in 14-3-3σ-depleted SNU-484(Mock) cells.

Since 14-3-3σ is known to enhance the chemosensitivity of some types of cancer cells [24-27], we first examined whether depletion of endogenous 14-3-3σ expression could enhance chemoresistance of gastric cancer cells. For this purpose, we used SNU-484(Mock) cells in which 14-3-3σ, which is highly expressed (Fig. 1B), was depleted by employing siRNA transfection. As shown in Fig. 2A, the expression of 14-3-3σ was markedly reduced in 14-3-3σ-specific siRNA transfected cells, but not in control siRNA transfected cells. To determine whether the depletion of 14-3-3σ expression affects cisplatin-induced apoptosis, we analyzed the cells by TUNEL staining. Control cells were highly sensitive to cisplatin-induced apoptosis (Fig. 2B and C), as previously reported.^22^ However, depletion of 14-3-3σ significantly attenuated cisplatin-induced apoptosis (Fig. 2B and C) and cleavage of poly(ADP-ribose) polymerase (PARP) (Fig. 2D) in SNU-484(Mock) cells. These results led us to hypothesize that the downregulation of 14-3-3σ contributes to the RhoGDI2-induced chemoresistance of gastric cancer cells.

### Ectopic expression of 14-3-3σ restores chemosensitivity to cisplatin in RhoGDI2-overexpressing gastric cancer cells

**Figure 3:**
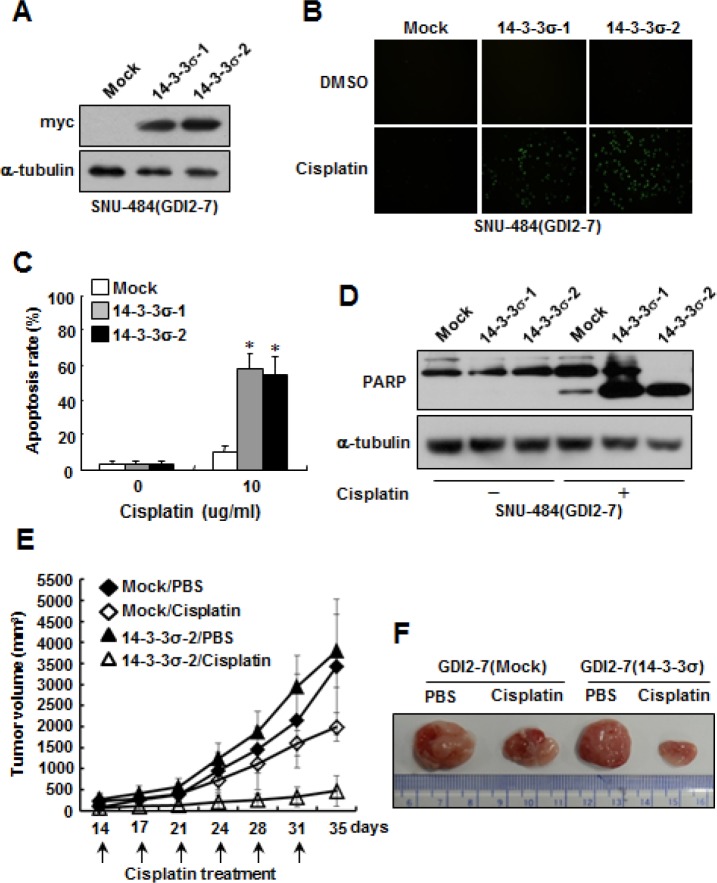
Ectopic expression of 14-3-3σ restores chemosensitivity to cisplatin in RhoGDI2-overexpressing gastric cancer cells (A) Representative immunoblot for myc in 14-3-3σ-overexpressing SNU-484(GDI2-7) cells. (B) Representative images for TUNEL staining of 14-3-3σ-overexpressing SNU-484(GDI2-7) cells after cisplatin treatment (10 μg/ml) for 24 h. (C) Histogram shows the ratio of TUNEL-positive 14-3-3σ-overexpressing SNU-484(GDI2-7) cells after cisplatin treatment for 24 h. Data are mean ± SD of three individual experiments, each in triplicate. *, *P* < 0.01 as determined by paired Student *t* test. (D) Representative immunoblot for PARP cleavage after cisplatin treatment in 14-3-3σ-overexpressing SNU-484(GDI2-7) cells. (E) *In vivo* chemoresistance assay of 14-3-3σ-overexpressing SNU-484(GDI2-7) cells. Xenograft tumor volumes in mice treated with cisplatin or PBS are shown. Tumor volume is presented as the mean ± SD (*n* = 12 mice in each group). (F) Representative images of tumor derived from 14-3-3σ-overexpressing SNU-484(GDI2-7) and control cells in mice treated with cisplatin or PBS for 35 days.

To determine whether 14-3-3σ expression can alter cellular sensitivity to cisplatin in RhoGDI2-overexpressing gastric cancer cells, we established 14-3-3σ-overexpressing cells in RhoGDI2-overexpressing SNU-484(GDI2-7) cells. To this end, a myc-tagged 14-3-3σ expression vector (pcDNA4-myc-His/14-3-3σ) or empty vector (pcDNA4-myc-His) was stably transfected into RhoGDI2-overexpressing SNU-484(GDI2-7) cells. The expression levels of 14-3-3σ in the respective cells were verified by performing western blot analysis using anti-myc antibody (Fig. 3A). RhoGDI2-overexpressing SNU-484(GDI2-7/Mock) control cells were highly resistant to cisplatin-induced apoptosis (Fig. 3B and C), as previously reported [22]. However, the overexpression of 14-3-3σ significantly increased cisplatin-induced apoptosis (Fig. 3B and C) and PARP cleavage (Fig. 3D) in RhoGDI2-overexpressing SNU-484(GDI2-7) cells. These results suggest that the downregulation of 14-3-3σ expression confers resistance to RhoGDI2-overexpressing gastric cancer cells against cisplatin-induced apoptosis.

To determine if 14-3-3σ expression affects the chemosensitivity of RhoGDI2-overexpressing gastric cancer cells *in vivo*, 14-3-3σ-overexpressing SNU-484(GDI2-7/14-3-3σ-2) cells and control cells (GDI2-7/Mock) were injected subcutaneously into the flanks of nude mice. Growth of the 14-3-3σ-overexpressing SNU-484(GDI2-7/14-3-3σ-2) cell-injected tumor did not differ from that of control cells (Fig. 3E and F). Cisplatin significantly inhibited tumor growth in mice that had been injected with 14-3-3σ-overexpressing SNU-484(GDI2-7/14-3-3σ-2) cells, but not in mice that had been injected with control cells (GDI2-7/Mock) (Fig. 3E and F). These findings indicate that 14-3-3σ contributes to the inhibition of resistance to cisplatin of RhoGDI2-expressing gastric cancer cells in xenograft tumor models.

### Suppression of Erk and p38 activities enhances cisplatin resistance of gastric cancer cells

**Figure 4:**
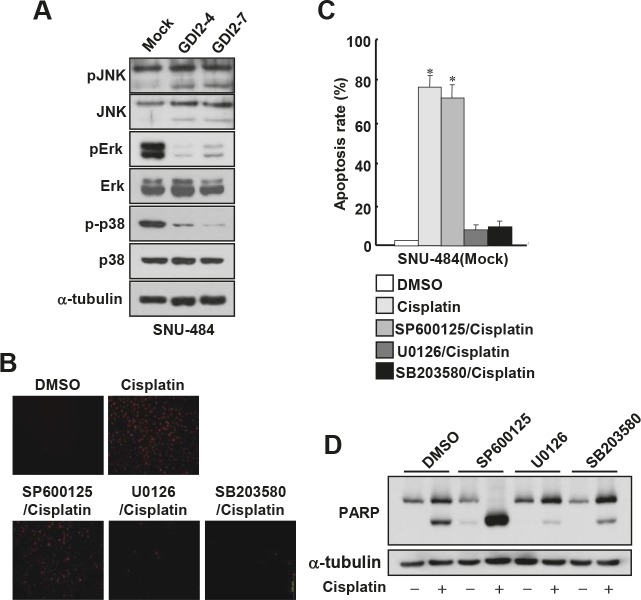
Suppression of Erk and p38 activation attenuates cisplatin-induced apoptosis in gastric cancer cells (A) Representative immunoblot for phosphorylated and total Erk, JNK, and p38MAPK in RhoGDI2-overexpressing SNU-484(GDI2-4 and GDI2-7) cells. (B) Representative images for TUNEL staining of SNU-484(Mock) cells in the presence or absence of respective inhibitor after cisplatin treatment (10 μg/ml) for 24 h. (C) Histogram shows the ratio of TUNEL-positive SNU-484(Mock) cells in the presence or absence of respective inhibitor after cisplatin treatment (10 μg/ml) for 24 h. Data are mean ± SD of three individual experiments, each in triplicate. *, *P* < 0.01 as determined by paired Student *t* test. (D) Representative immunoblot for PARP cleavage of SNU-484(Mock) cells in the presence or absence of respective inhibitor after cisplatin treatment.

Since mitogen-activated protein kinase (MAPK) pathways are implicated in the execution of apoptosis by different cytotoxic agents and p38/JNK kinase activities have been known to be increased in RhoGDI2-depleted breast cancer cells [15], we first assessed whether the activities of these apoptosis-related kinases are suppressed in RhoGDI2-overexpressing gastric cancer cells. To this end, we examined MAPK activation in RhoGDI2-overexpressing SNU-484 cells by assessing their phosphorylation states by using antibodies specific to the phosphorylated species of each enzyme. As shown in Fig 4A, the phosphorylation levels of Erk and p38, but not JNK, were significantly decreased in RhoGDI2-overexpressing SNU-484(GDI2-4 and GDI2-7) cells compared with control cells (Mock) under normal culture condition. We next examined whether suppression of Erk or p38 activity affects cisplatin-induced apoptosis in gastric cancer cells. Suppression of Erk and p38 activity by U0126 and SB203580, respectively, significantly inhibited cisplatin-induced apoptosis (Fig. 4B and C) and PARP cleavage (Fig. 4D) in SNU-484(Mock) cells. These results suggest that suppression of Erk and p38 activity is critical for the cisplatin resistance of gastric cancer cells.

### Ectopic expression of 14-3-3σ attenuates RhoGDI2-induced cisplatin resistance of gastric cancer cells through Erk and p38 activation

**Figure 5:**
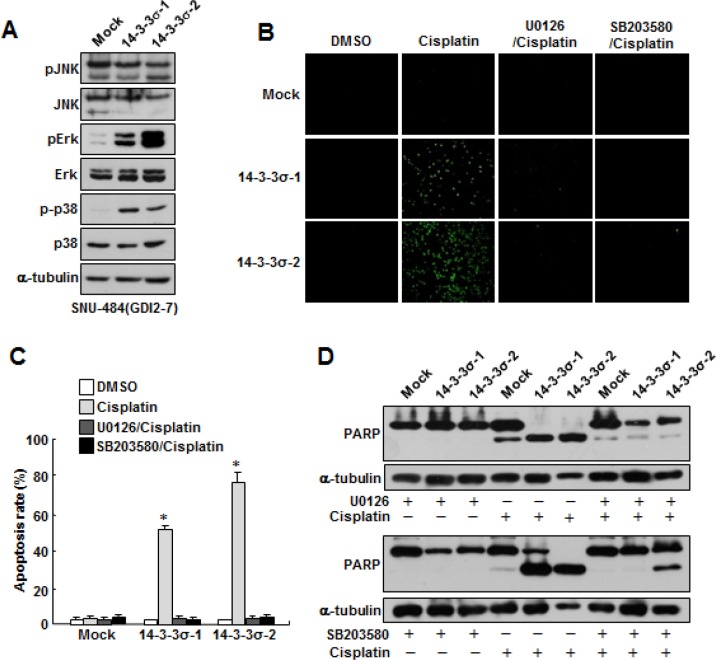
Ectopic expression of 14-3-3σ attenuates RhoGDI2-induced cisplatin resistance in gastric cancer cells through Erk and p38 activation (A) Representative immunoblot for phosphorylated and total MAPK in 14-3-3σ-restored RhoGDI2-overexpressing SNU-484(GDI2-7) cells. (B) Representative images for TUNEL staining of 14-3-3σ-restored RhoGDI2-overexpressing SNU-484(GDI2-7) cells in the presence or absence of respective inhibitor after cisplatin treatment (10 μg/ml) for 24 h. (C) Histogram shows the ratio of TUNEL-positive 14-3-3σ-restored RhoGDI2-overexpressing SNU-484(GDI2-7) cells in the presence or absence of respective inhibitor after cisplatin treatment (10 μg/ml) for 24 h. Data are mean ± SD of three individual experiments, each in triplicate. *, *P* < 0.01 as determined by paired Student *t* test. (D) Representative immunoblot for PARP cleavage of 14-3-3σ-restored RhoGDI2-overexpressing SNU-484(GDI2-7) cells in the presence or absence of respective inhibitor after cisplatin treatment.

Since 14-3-3σ regulates MAPK activity and the activities of Erk and p38 were repressed in RhoGDI2-overexpressing SNU-484(GDI2-4 and GDI2-7) cells (Fig. 4A), we examined whether the downregulation of 14-3-3σ expression is critical for the repression of Erk and p38 activities in RhoGDI2-overexpressing SNU-484(GDI2-7) cells. As we expected, the levels of phospho-Erk and phospho-p38 markedly increased in 14-3-3σ-overexpressing SNU-484(GDI2-7) cells (14-3-3σ-1 and 14-3-3σ-2) compared with control (Mock) cells (Fig. 5A). As shown in Figure 3, the overexpression of 14-3-3σ significantly increased cisplatin-induced apoptosis in RhoGDI2-overexpressing SNU-484(GDI2-7) cells. We next examined whether 14-3-3σ-mediated activation of Erk and p38 activities is critical for the recovery of cisplatin sensitivity in RhoGDI2-overexpressing SNU-484(GDI2-7) cells. Suppression of Erk and p38 activities by their specific inhibitors, respectively, markedly inhibited cisplatin-induced apoptosis (Fig. 5B and C) and PARP cleavage (Fig. 5D) in 14-3-3σ-overexpressing SNU-484(GDI2-7) cells (14-3-3σ-1 and 14-3-3σ-2). All of these results suggest that the suppression of 14-3-3σ-mediated Erk and p38 activation is critical for the cisplatin resistance of RhoGDI2-expressing gastric cancer cells.

### 14-3-3σ suppresses RhoGDI2-induced migration and invasion abilities of gastric cancer cells

**Figure 6:**
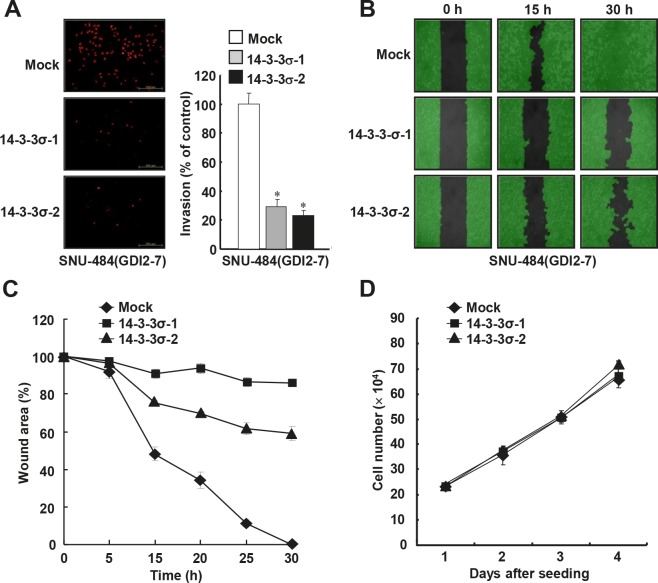
Ectopic expression of 14-3-3σ suppresses RhoGDI2-induced gastric cancer cell migration and invasion (A) Representative images of invading cells through the matrigel-coated membrane stained by propidium iodide. Quantitative data of invasion assay are expressed relative to the invasion ability of SNU-484(GDI2-4/Mock) cells. Data are mean ± SD of three individual experiments, each in triplicate. *, *P* < 0.01 as determined by paired Student *t* test. (B) Representative images of migrating cells obtained at indicated time points after wound formation by phase contrast microscopy and using Wimasis Image Analysis software. (C) Quantitative analysis of wound healing assay by using the WimScratch software (Wimasis). Data represent the percentage of wound area at indicated time points in control and 14-3-3σ-overexpressing SNU-484(GDI2-4) cells. (D) Effect of 14-3-3σ overexpression on the proliferation of RhoGDI2-overexpressing SNU-484(GDI2-7) cells.

Since RhoGDI2 promotes gastric cancer cell invasion^21^ as well as enhances cisplatin resistance, we next examined whether the restoration of 14-3-3σ could alter the migration and invasive properties in RhoGDI2-overexpressing gastric cancer cells. Ectopic expression of 14-3-3σ considerably decreased the invasiveness of RhoGDI2-overexpressing SNU-484(GDI2-7) cells (14-3-3σ-1 and 14-3-3σ-2) compared with control (Mock) cells (Figure 6A). Next, we checked the migrating ability of 14-3-3σ-overexpressing SNU-484(GDI2-7) cells by using a wound healing assay. For this analysis, we used a culture-insert, where a non-bias cell-free gap is produced as the “wound” when the culture-insert is removed. Immediately after removal of the culture-insert, cell images were obtained at various time points (0-30 h) under a light microscope. After 30 h, complete wound closure (100%) was achieved in the RhoGDI2-overexpressing SNU-484 control (Mock) cells, whereas only 14.9% (14-3-3σ-1) and 41% (14-3-3σ-2) wound closure was achieved in 14-3-3σ-overexpressing SNU-484(GDI2-7) cells compared with the control cells (Figure 6B and C). Taken together, these results suggest that the downregulation of 14-3-3σ expression plays a key role in RhoGDI2-induced gastric cancer cell migration and invasion. In an effort to exclude the possibility that the effect of 14-3-3σ on the migration and invasion of RhoGDI2-overexpressing gastric cancer cells was attributable to different proliferation rates, we compared the growth rates of 14-3-3σ-overexpressing SNU-484(GDI2-7) cells (14-3-3σ-1 and 14-3-3σ-2) with those of control (Mock) cells. Under the same growth conditions, all of the cells exhibited similar growth rates (Figure 6D), thereby indicating that decreased tumor cell migration and invasion via the expression of 14-3-3σ in the respective cells was not associated with their proliferation rates.

## DISCUSSION

Several studies over the last decade have linked RhoGDI1 expression to apoptosis and chemoresistance of various human cancer cells. For example, the reduction of RhoGDI1 expression is associated with astrocytoma cell protection and tamoxifen resistance of breast cancer cells [28, 29]. In addition, Ronneburg et al. demonstrated that RhoGDI1 may sensitize invasive breast cancer to treatment with CMF (cyclophosphamide, methotrexate, and 5-fluorouracil), and higher RhoGDI1 expression tends to be correlated with a better clinical outcome [30]. However, RhoGDI1 was also described as an antiapoptotic protein in breast, lymphoma, fibrosarcoma, and lung cancer cells [31-33]. In contrast to RhoGDI1, several groups have focused their research on elucidating the explicit mechanisms by which RhoGDI2 regulates aggressive features of cancer cells, particularly motility, invasiveness, and metastasis. However, we recently suggested that RhoGDI2 enhances the chemoresistance of gastric cancer through the upregulation of Bcl-2 expression as well as promotes tumor growth and malignant progression.^22^ Consistent with our results, Zheng et al. suggested that the knockdown of RhoGDI2 expression significantly increases the sensitivity of colon cancer cells to 5-FU [34], and that the ectopic expression of RhoGDI2 in gastric cancer cells induces resistance to 5-FU and reverses 5-FU-induced G2/M phase arrest [35].

RhoGDI1 protects breast cancer cells from drug-induced apoptosis through the inhibition of Rac1 cleavage that is mediated by capase-3 [31]. Although RhoGDI1 is completely resistant to degradation during apoptosis, RhoGDI2 is well characterized as being a substrate for caspases and is cleaved in various cells during apoptosis [36]. Therefore, RhoGDI2 may act as an antiapoptotic molecule via a mechanism that is distinct from RhoGDI1 and may do so prior to caspase activation during drug-induced apoptosis. To delineate the mechanism by which RhoGDI2 contributes to chemoresistance and tumor metastasis, we performed 2-DE on proteins that were derived from a RhoGDI2-overexpressing SNU-484 human gastric cancer cell line and control cells. We found that the expression levels of 14-3-3σ were significantly downregulated in the RhoGDI2-overexpressing gastric cancer cells [23]. The results of this study indicate that 14-3-3σ is a direct target of RhoGDI2 and that the downregulation of 14-3-3σ is important for enhancing the chemoresistance of RhoGDI2-expressing gastric cancer cells. 14-3-3σ was first identified as being a human mammary epithelium-specific marker 1 (HME1) and participates in the regulation of subcellular localization, protein stability, cell apoptosis, proliferation, and the cell cycle [37-39]. However, 14-3-3σ is directly associated with human cancers, and the downregulation of 14-3-3σ expression has been observed in various human cancers, including those of the lung, prostate, breast, and liver [40, 41]. Consistent with our findings, several previous findings have shown that 14-3-3σ expression is elevated in response to different cellular stresses, and that it enhances the chemosensitivity of human colorectal, breast, and nasopharyngeal cancer [24-27].

We also suggested that the ectopic expression of 14-3-3σ could activate Erk and p38 MAPK, and that suppression of 14-3-3σ-mediated Erk and p38 activation is critical for the cisplatin resistance of RhoGDI2-overexpressing gastric cancer cells. We showed that the phosphorylation levels of Erk and p38 kinase are markedly downregulated in RhoGDI2-overexpressing (14-3-3σ downregulated) gastric cancer cells. However, the restoration of 14-3-3σ expression reverses this phenomenon. It is believed that MAPK activation is a major component that decides the fate of a cell in response to cisplatin. The pro-death or pro-survival roles of MAPKs in response to cisplatin could depend on the type of activated MAPK. While the activation of p38 plays only a pro-death role, the induction of Erk can take on both roles (survival or cell death) as a consequence [42]. Consistent with our results, the knockdown of RhoGDI2 expression significantly increases the phosphorylation levels of p38 kinase, and pretreatment with p38 kinase inhibitor significantly inhibits the apoptosis of RhoGDI2-depleted breast cancer cells [15]. Benzinger et al. also showed that the ectopic expression of 14-3-3σ enhances Erk1/2 activity in colon cancer cells [43]. The increase in MAPK activity that was observed after 14-3-3σ expression may be due to interactions with multiple ligands. Among others, A-RAF, B-RAF, and c-RAF seem to be good candidates because previous studies on the interaction between RAF proteins and 14-3-3 isoforms have shown that 14-3-3 proteins are critical modulators of RAF activity [44].

We also examined the possible pathways that lead to RhoGDI2-induced 14-3-3σ downregulation. Recently, we demonstrated that phospholipase C-gamma (PLCγ) is activated in RhoGDI2-overexpressing SNU-484 cells, and that it is required for RhoGDI2-mediated cisplatin resistance and cancer cell invasion in gastric cancer [45]. To determine whether PLC is required for RhoGDI2-induced 14-3-3σ downregulation, we examined the expression levels of 14-3-3σ in PLCγ-depleted SNU-484(GDI2) and control cells, but did not observe any difference (data not shown). Alternatively, ongoing studies in our laboratory have revealed that Rac1, but not RhoA or Cdc42, is activated in RhoGDI2-overexpressing gastric cancer cells. Therefore, we are now examining whether the activation of Rac1 is involved in RhoGDI2-induced 14-3-3σ downregulation.

## MATERIALS AND METHODS

### Cell cultures and reagents

Human gastric cancer cell lines SNU-484 derived RhoGDI2-overexpressing cells (GDI2-4 and GDI2-7) were maintained in RPMI-1640 medium (SIGMA). Human cervical cancer cell line HeLa and breast cancer cell line MCF-7 were cultivated in DMEM (SIGMA). All cell lines were maintained as mono-layer cultures in each optimal medium supplemented with 10% heat-inactivated FBS (Gibco, Invitrogen) and 2% of a penicillin-streptomycin (antibiotic-antimycotic) mixture (Gibco, Invitrogen) The SNU-484 cells stably transfected with RhoGDI2 and MKN-28 cells stably transfected with shRNA-expressing lentiviral vector for targeting RhoGDI2 were described in our previous report [21, 22]. Cisplatin was purchased from SIGMA. SP600125 (JNK inhibitor), U0126 (MEK1/2 inhibitor), and SB203580 (p38 inhibitor) were purchased from Cell Signaling Technology.

### Reverse Transcription-PCR analysis

Total RNA was isolated using an RNeasy mini kit (Qiagen, Valencia, CA, USA) according to the manufacturer's instructions. RT-PCR was performed using a Maxime RT-PCR PreMix kit (Intron, Taejon, Korea). 200 ng of total RNA and specific primer were added into the Maxime RT-PCR PreMix tubes and RNase-free water was added to a total volume of 20 μl. RT-PCR was performed using a Thermo Electron PCR thermal cycler. Amplified products were separated on 1~1.5% agarose gels. The RT-PCR conditions were as follows: 45°c for 30 min (reverse transcription), 94°c for 5 min (inactivation of RTase), 94°c for 1 min, 55°c for 1 min, 72°c for 1 min for 23~38 cycles, followed by 10 min of incubation at 72°c. The used primers were described in Supplementary Table 1.

### Construction of the 14-3-3σ expression plasmid and transfection

Human 14-3-3σ cDNA was amplified by PCR using the following primers: 5'-GATCGGAATCCAGAGCGAAACCTGCTCTCAG-3' and 5'-GATCGGGATCCTGATGAGGGTGCTGTCTTTG-3'. PCR products were cloned into the pcDNA4/myc-His^©^ (Invitrogen). RhoGDI2-overexpressing SNU-484(GDI2-7) cells were transfected with 14-3-3σ expressing plasmid by using the FuGENE^®^ 6 reagent (Promega) following the manufacturer's instructions. After 48h of incubation, cells were treated with Zeocin™ (100 ug/ml) for selection. For transient transfection, 3 × 10^5^/ml HeLa and MCF-7 cells were seeded in 6-well plate for 24 h and transfected with the indicated plasmids by using FuGENE^®^ HD reagent (Promega). After 48 h, the cells were harvested and analyzed by western blot.

### Antibodies and western blot analysis

Rabbit anti-PARP, anti-SAPK/JNK, anti-Phospho-SAPK/JNK (Thr183/Tyr185), anti-p44/42 Map Kinase, anti-Phospho-p44/42 Map Kinase (Thr202/Tyr204), anti-p38 MAP Kinase, and anti-Phospho-p38 MAP Kinase (Thr180/Tyr182) antibodies were purchased from Cell Signaling Technology. Anti-14-3-3 sigma antibody was purchased from Thermo Scientific. Mouse anti-α-tubulin and anti-Flag antibodies were purchased from Sigma. Mouse anti-myc antibody was purchased from IGTHERAPY. For western blot analysis, cells were harvested after defined time and lysed in lysis buffer (20 Mm Tris (pH 7.4), 2 mM EDTA, 150 mM sodium chloride, 1 mM sodium deoxycholate, 1% Triton X-100, 10% glycerol, 2 pills protease inhibitor cocktail (Roche)) on ice for 1 h and centrifuged at 13,000 rpm for 15 min. Cell lysates were separated by 8–12% SDS-PAGE and transferred to a polyvinylidene difluoride membrane (Amersham Bioscience). Subsequently, the membrane was incubated in TBST supplemented with 5% non-fat dry milk and probed with the appropriate primary antibodies. The bound antibodies were visualized with a suitable secondary antibody conjugated with horseradish peroxidase using enhanced chemiluminescence (ECL) reagent WESTSAVE up (AbFRONTIER, Korea).

### RNA interference experiments

Two different siRNA oligo duplexes for targeting 14-3-3σ were purchased from Bioneer (Daejeon, Korea). The sequence was as follows; si14-3-3σ-1: 5'-GGAUCCCACUCUUCUUGCA-3', si14-3-3σ-2: 5'-GACCAUGUUUCCUCUCAAU-3'. Transient transfection of siRNA oligo duplex was accomplished using siLentFect™ Reagent (BIO-RAD) followed by instructions of manufacturers. After incubation for 48hrs, the cells were harvested and efficiency of each siRNA oligo duplex was confirmed by western blotting using anti-14-3-3σ antibody.

### Apoptosis detection

Apoptosis was measured by the terminal deoxynucleotidyl transferase-mediated deoxyuridine triphosphate nick-end labeling (TUNEL) assay using the In Situ Cell Death Detection Kit, Fluorescein or TMR red (Roche Applied Science, Germany) following the manufacturer's instruction. Cisplatin-treated or non-treated cells were washed with cold PBS and fixed with 4% paraformaldehyde. Fixed cells were permeabilized and stained using the TUNEL reaction mixture in the dark. The cells were then stained with 1 μg/ml DAPI solution for 5 min at room temperature in the dark and observed under a fluorescence microscope. The apoptosis rate was quantified by the TUNEL-positive rate.

### Tumorigenicity in nude mice

For tumorigenicity experiments, six-week-old female BALB/cSlc-nude mice were injected subcutaneously with 5.5×10^6^ SNU-484(GDI2-7/14-3-3σ-2) or SNU-484(GDI2-7/Mock) cells. When tumors measured an average volume of 50 mm^3^, the mice (12 per group) were treated with cisplatin (5mg/kg, 2 times a week) or physiological saline for three weeks. Tumors were measured with calipers to estimate volume(0.5×width^2^×length). All animal experiments were approved by the Institutional Animal Care and Use Committees (IACUC) of Gyeongsang National University and followed National Research Council Guidelines.

### Invasion and migration assay

The invasion ability of cancer cells was assessed using a matrigel-based transwell system. Briefly, 24-well cell culture plate inserts with 8-μm pore size polycarbonate membrane (Corning, NY, USA) were precoated with 100 μl matrigel/DMEM solution (2.2 mg/ml, BD Bioscience, Bedford, MA) and incubated at 37°c for 2 h or overnight at 4°c. All the cells were preincubated in serum-free media with or without inhibitors for 24 h. 2.5 × 10^5^ cells in 250 μl of medium (no serum) were placed in the insert and allowed to invade for 48 h. The lower chamber was filled with 750 μl of appropriated media containing 20% FBS. After incubation, medium remaining on top of the insert were removed by pipetting and non-invading cells on the upper surface of the insert membrane were removed with cotton swab. After washing twice with PBS, the insert membranes were fixed for 10 min with MeOH/Acetic acid (3:1) at −20°c and stained with 50 μg/ml propidium iodide (SIGMA) for 20 min at 37°c. The upper surface of the insert membrane was gently scrubbed with cotton swab again and washed with distilled water. Membranes were cut and mount on slide glass and the number of invaded cells was counted microscopically at 100-200 × magnification. For wound healing assays, 4.9 × 10^4^ cells in 70 μl of medium were seeded into Culture-Insert (Ibidi, Munich, Germany). After the cells were confluent, to inhibit the effect of cell proliferation, the cells were pretreated with 10 μg/ml mitomycin C (SIGMA) for 2 h, and washed with culture medium. After removal of Culture-Insert, cells were incubated with fresh media and photographs of the migration assay were taken at 0, 5, 15, 20, 25 and 30 h using a phase-contrast microscope with digital camera. The cell migration was quantified by calculating the cell-covered area using WimScratch software (Wimasis, Munich, Germany).

### Proliferation assay

The cells were placed in a 6-well plate at a concentration of 3 × 10^4^ cells per well. After incubation for 1 to 4 days, cells were trypsinized and resuspended in 3 ml of appropriate medium. Cell suspensions were centrifuged at 1000 rpm for 5 min. Cell pellets were resuspended in 1 ml of appropriate medium. The viable cells were counted with a hemocytometer after trypan blue staining.

### Statistical analysis

We performed statistical analysis using the unipolar, paired Student *t*-test. The significance of the data was accepted when the *P* value was less than 0.05.

## Supplementary Table


