# Supplementary material for: 14-3-3σ attenuates RhoGDI2-induced cisplatin resistance through activation of Erk and p38 in gastric cancer cells
Source: Oncotarget. 2013 Oct 19;4(11):2045–56. doi: 10.18632/oncotarget.1334 (PMC3875768; doi:10.18632/oncotarget.1334)
Supplement: Supplementary file 1 [file oncotarget-04-2045-s001.ppt]

## Slide 1
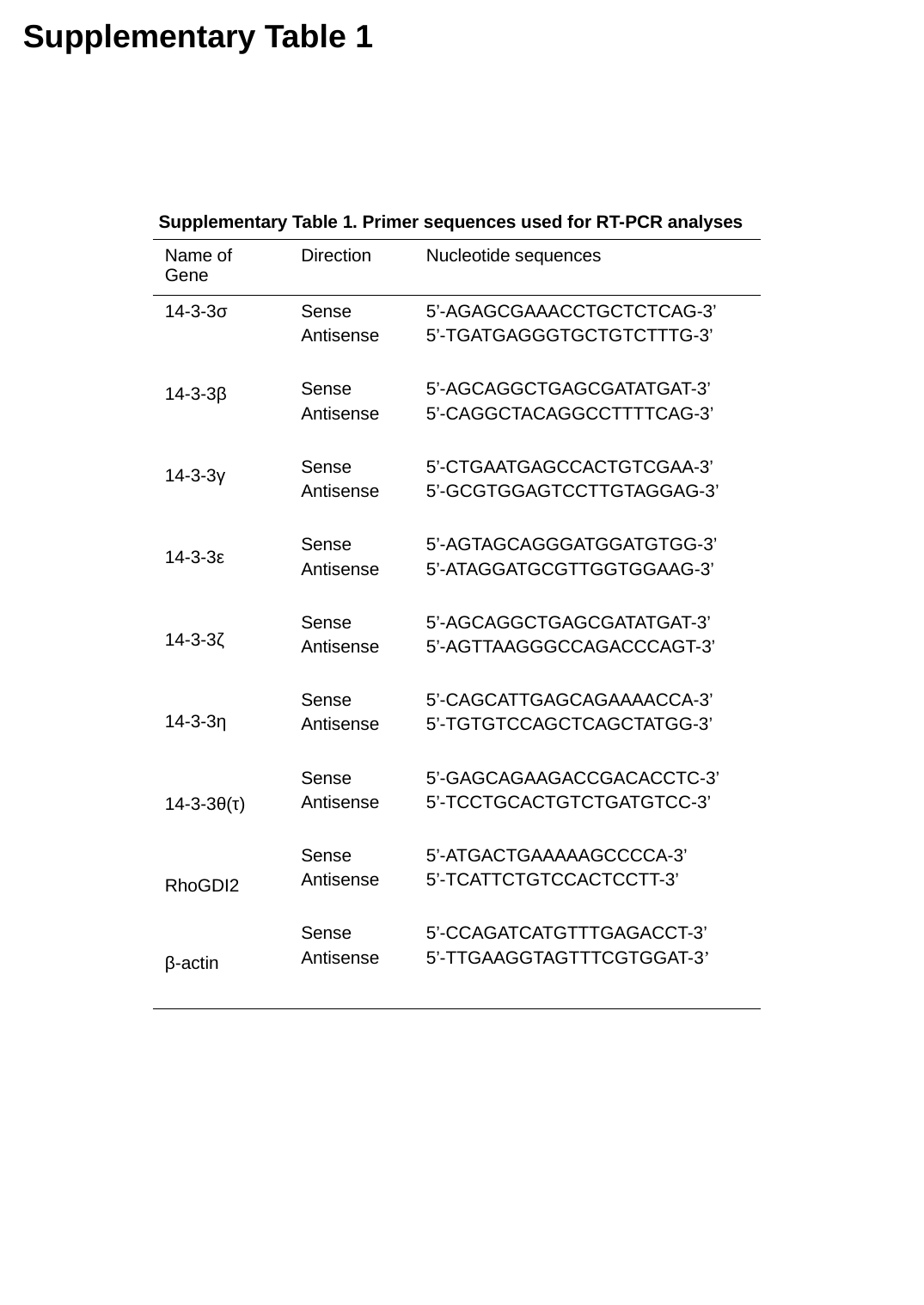

Supplementary Table 1
Supplementary Table 1. Primer sequences used for RT-PCR analyses
| Name of Gene | Direction | Nucleotide sequences |
| --- | --- | --- |
| 14-3-3σ 14-3-3β 14-3-3γ 14-3-3ε 14-3-3ζ 14-3-3η 14-3-3θ(τ) RhoGDI2 β-actin | Sense Antisense Sense Antisense Sense Antisense Sense Antisense Sense Antisense Sense Antisense Sense Antisense Sense Antisense Sense Antisense | 5’-AGAGCGAAACCTGCTCTCAG-3’ 5’-TGATGAGGGTGCTGTCTTTG-3’ 5’-AGCAGGCTGAGCGATATGAT-3’ 5’-CAGGCTACAGGCCTTTTCAG-3’ 5’-CTGAATGAGCCACTGTCGAA-3’ 5’-GCGTGGAGTCCTTGTAGGAG-3’ 5’-AGTAGCAGGGATGGATGTGG-3’ 5’-ATAGGATGCGTTGGTGGAAG-3’ 5’-AGCAGGCTGAGCGATATGAT-3’ 5’-AGTTAAGGGCCAGACCCAGT-3’ 5’-CAGCATTGAGCAGAAAACCA-3’ 5’-TGTGTCCAGCTCAGCTATGG-3’ 5’-GAGCAGAAGACCGACACCTC-3’ 5’-TCCTGCACTGTCTGATGTCC-3’ 5’-ATGACTGAAAAAGCCCCA-3’ 5’-TCATTCTGTCCACTCCTT-3’ 5’-CCAGATCATGTTTGAGACCT-3’ 5’-TTGAAGGTAGTTTCGTGGAT-3’ |
